# Evaluation of Antibacterial and Antibiofilm Properties of Phenolics with Coumarin, Naphthoquinone and Pyranone Moieties Against Foodborne Microorganisms

**DOI:** 10.3390/molecules30040944

**Published:** 2025-02-18

**Authors:** Alejandra Alejo-Armijo, Antonio Cobo, Alfonso Alejo-Armijo, Joaquín Altarejos, Sofía Salido, Elena Ortega-Morente

**Affiliations:** 1 Department of Health Sciences, Faculty of Experimental Sciences, University of Jaén, Campus of International Excellence in Agri-Food (ceiA3), 23071 Jaén, Spain; ajanja00@gmail.com (A.A.-A.); acmolinos@ugr.es (A.C.); 2 Department of Inorganic and Organic Chemistry, Faculty of Experimental Sciences, University of Jaén, Campus of International Excellence in Agri-Food (ceiA3), 23071 Jaén, Spain; jaltare@ujaen.es (J.A.); ssalido@ujaen.es (S.S.)

**Keywords:** procyanidin analogs, antibacterial activity, antibiofilm activity, coumarin, naphthoquinone, foodborne bacteria

## Abstract

Numerous studies have previously demonstrated the antimicrobial activity of plant extracts rich in procyanidins. However, these investigations that focused on uncharacterized extracts do not provide information on the structure–activity relationships of these compounds. The aim of this work was to investigate the antibacterial and antibiofilm properties of 27 phenolics with coumarin, naphthoquinone and pyranone moieties against foodborne microorganisms, as well as to establish structure–activity relationships. Minimal inhibitory concentrations (MICs) for each compound were investigated, as well as their ability for inhibiting biofilm formation as well as disrupting previously formed biofilms by food pathogens. Our compounds show high antibacterial and antibiofilm activities against Gram-positive bacteria. Regarding the structure–activity relationships observed, the coumarin moiety seems to favor the antibacterial activity against both *S. aureus* strains assayed, while a naphthoquinone moiety enhances antibacterial effects against *B. cereus*. Moreover, the replacement of OH groups in the B-ring by methoxy groups impairs antibacterial activity of the compounds against target bacteria, while the presence of Cl or OH groups in the molecules seems to enhance the inhibition of biofilm formation as well as the disruption of preformed biofilms. These results may be of great relevance for the food sector, increasing the options of additives that can be used industrially.

## 1. Introduction

Procyanidins (PCs) are ubiquitously present in a wide range of foods and crop plants, such as cranberry, cocoa, apple or grape [1,2,3]. However, the PC composition varies significantly across different plant species, due to variations in the configuration of main moieties, the types of bonds between units, and the varying substituents in the molecules [4]. Numerous studies have evaluated the antimicrobial activity of plant extracts rich in procyanidins and their impact on the inhibition of urinary tract and oral infections, bacterial adhesion, and biofilm formation [1,3,5]. However, most publications only evaluate the antimicrobial potential of crude extracts [6], and only a few of these studies have established correlations between the structural attributes and their biological activities [7,8].

The antimicrobial activity of these compounds is mainly based on metabolic disruption, DNA interactions or osmotic imbalance, while their antibiofilm activities include antiadhesive effects but also the modulation of mobility and quorum sensing [9]. PCs can also potentiate the activities of antibiotics and antifungals in diverse ways, exerting a synergistic effect with beta-lactams against *Enterobacteriaceae* clinical isolates, as well as against extended-spectrum β-lactamases (ESBLs) and metallo-β-lactamases producing *E. coli*, as well as against carbapenemases producing human pathogens [10], which makes them valuable tools in the prevention and treatment of urinary tract infections (UTIs) [11].

**Figure 1:**
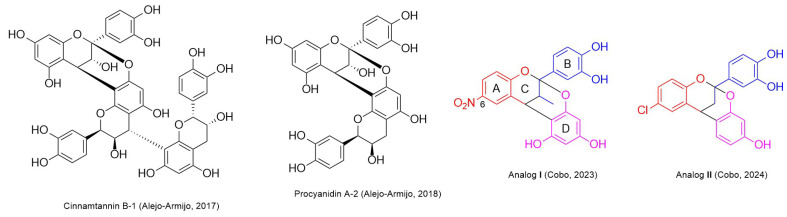
Natural A-type procyanidins (cinnamtannin B–1, procyanidin A–2) and some synthetic analogs to them (analogs **I** and **II**) previously studied by the authors [12,13,14,15]. Letters A–D refer to the type of ring in the structure.

We have also previously described desirable antibacterial and antibiofilm activities against foodborne bacteria by A-type procyanidins (PCs), a class of natural products from the condensed tannin family, and two main series of analogs related to them. Thus, once the A-type PC called cinnamtannin B–1 proved to be more effective than procyanidin B–2 (a B-type PC) [12], we synthesized several A-type analogs to procyanidin A–2, the structurally simplified version of cinnamtannin B–1, finding that the presence of a nitro (NO_2_) group at carbon 6 afforded higher activities [13] (Figure 1). Then, seven compounds with a NO_2_ group at C–6 and a variable number of OH groups on rings B and D, with or without a methyl group at the C-ring, were synthesized and evaluated. Among them, analog **I** proved to be more active regarding the inhibition of biofilm formation and disruption of preformed biofilms [14] (Figure 1). Taking into account that the presence of an electron-withdrawing group (NO_2_) on the A–ring seemed to improve the antibacterial and antibiofilm activities, eight additional analogs with chloro and bromo atoms at C–6 of the A-ring were synthesized and evaluated as potential biocides [15]. The results from this study showed that halogenated analogs (like analog **II**) were more active than the nitro derivatives previously reported (Figure 1).

**Figure 2:**
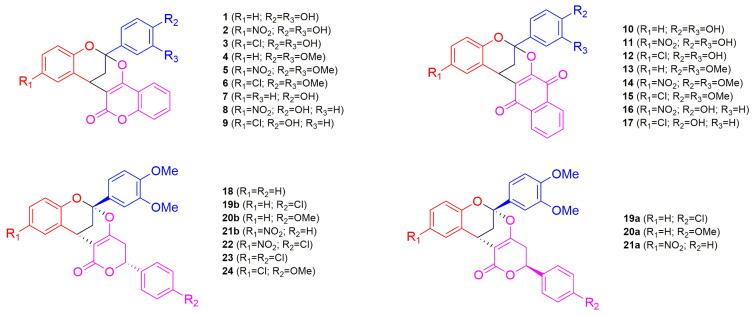
Analogs to A-type procyanidins, related to the leading structures of compounds **I** and **II** (Figure 1), evaluated in this work against foodborne pathogens. The synthesis of these compounds has been previously reported by us [16].

Continuing this work, we report herein on the evaluation of the antibacterial and antibiofilm properties of 27 compounds related to analogs **I** and **II** with coumarin, naphthoquinone and pyranone moieties (instead of phloroglucinol or resorcinol at D-ring) (Figure 2) against foodborne microorganisms, in an attempt to advance our knowledge on structure–activity relationships.

Among the target bacteria used in this study, the most susceptible strains were Gram-positive strains (*S. aureus* and *B. cereus* strains). Regarding the structure–activity relationships observed, a coumarin moiety seems to favor the antibacterial activity against *S. aureus* strains, while the naphthoquinone moiety enhances antibacterial effects against *B. cereus*. Moreover, the replacement of OH groups in the B-ring by methoxy groups impairs antibacterial activity of the compounds against target bacteria, while the presence of Cl or OH groups in the molecules seems to enhance the inhibition of biofilm formation as well as the disruption of preformed biofilms.

## 2. Results

### 2.1. Chemistry

**Scheme 1:**
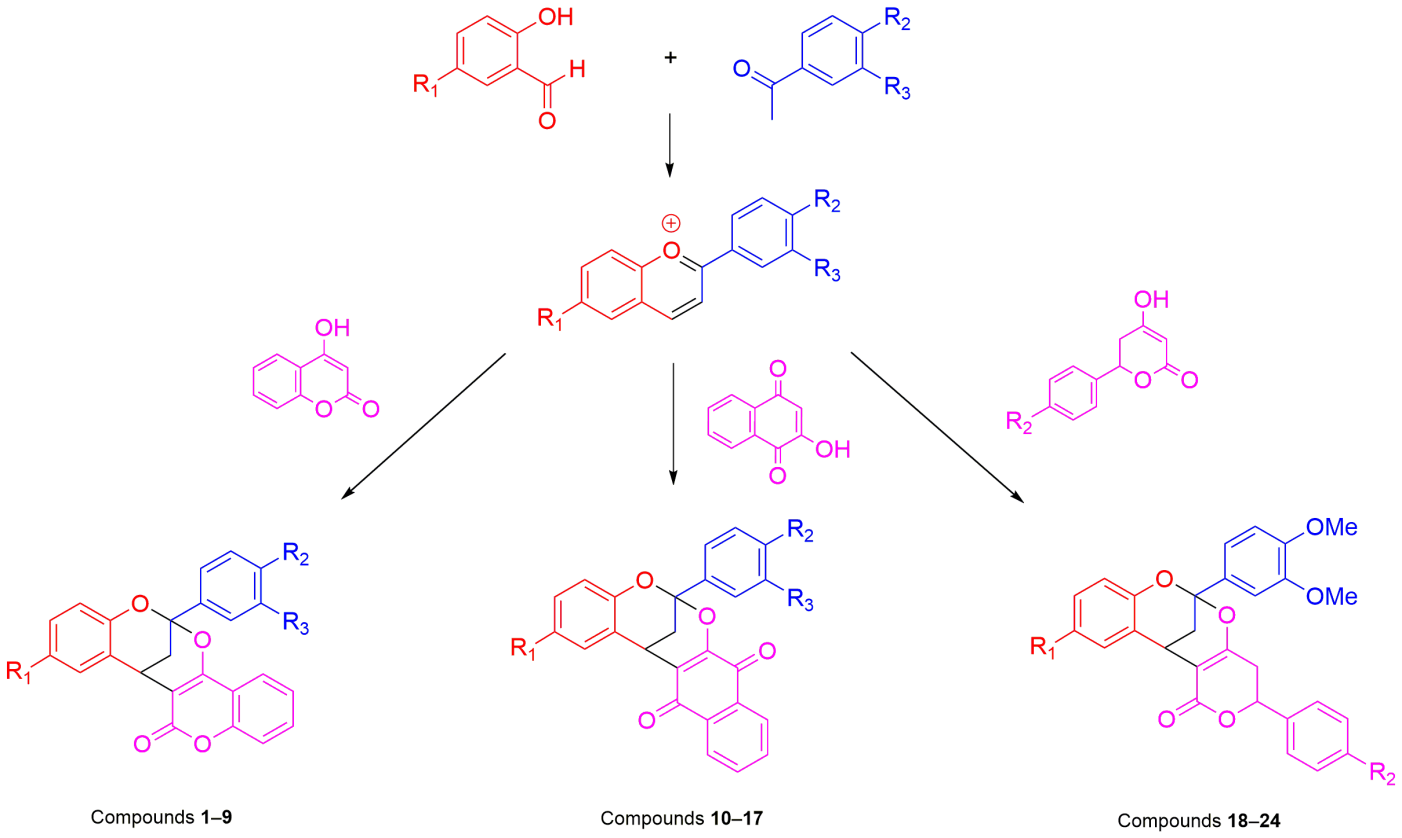
Synthesis of compounds **1**–**24** by reaction of flavylium salts with π-nucleophiles [16].

Compounds **1**–**24** (Table S1, Supplementary Materials) displayed in Figure 2 were obtained by reaction of flavylium salts with three different *π*-nucleophiles, such as 4-hydroxy-coumarin, 2-hydroxy-naphthoquinone and several pyranone derivatives (Scheme 1) [16]. Flavylium salts were synthesized by aldol condensation under acidic conditions between salicylic aldehyde and acetophenone derivatives according to procedures previously used by us [17].

### 2.2. Antibacterial Activity

As targets for these assays, eight strains from Type-Culture, belonging to genera *Staphylococcus*, *Listeria*, *Escherichia* and *Salmonella*, as well as twelve strains of our own collection of resistant strains from organic foods, both Gram-positive and Gram-negative ones, were used.

According to the preliminary standard agar diffusion assay, which showed eight potential susceptible strains and eleven active compounds (Table 1), minimal inhibitory concentration (MIC) values for each compound were obtained (Table 2).

These assays showed three susceptible strains (*S. aureus* CECT 976, *S. aureus* CECT 828 and *B. cereus* UJA 27q), with MIC values for various compounds assayed between 25 and 50 μg/mL against them.

**Figure 3:**
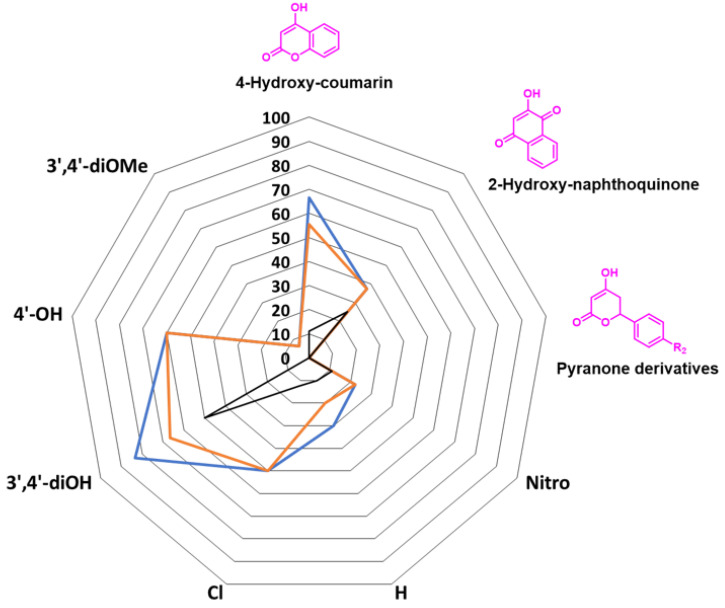
Structure–activity relationship of analogs to A-type procyanidins (**1**–**24**) against selected foodborne pathogens (*S. aureus* CECT 976, *S. aureus* CECT 828 and *B. cereus* UJA 27q). **Blue line**: number of compounds (expressed in percentage) with an MIC lower than 50 μg/mL against *S. aureus* CECT 976. **Orange line**: number of compounds (expressed in percentage) with an MIC lower than 50 μg/mL against *S. aureus* CECT 828. **Black line**: number of compounds (expressed in percentage) with an MIC lower 50 μg/mL against *B. cereus* UJA 27q.

Analogs **1**, **3**, **6**, **7**, **8**, **9** and **10** showed MIC values of 25 µg/mL against *S. aureus* CECT 976, while analogs **11** and **12** were able to inhibit the growth of this strain at 50 µg/mL. Thus, the presence of a coumarin moiety seems to favor the antibacterial activity against this strain, as shown in Figure 3.

Lowest MICs against *S. aureus* CECT 828 were found for analogs **3**, **7**, **9** and **11** (25 µg/mL). On the other hand, analogs **6**, **8**, **10** and **12** showed MICs of 50 µg/mL against these bacteria. The presence of a coumarin unit in the chemical structure seems to enhance again the antibacterial activity against these Gram-positive bacteria (Figure 3).

Analogs **3**, **10** and **11** showed MICs of 25 µg/mL against *B. cereus* UJA 27q. Most of the active analogs against these bacteria have a naphthoquinone moiety in their structure, which seems to increase the antibacterial activity against this Gram-positive bacillus (Figure 3).

*S. aureus* CECT 976 was the most susceptible strain among those studied, as nine analogs were able to inhibit the growth of this strain at a concentration of 25 µg/mL. *S. aureus* CECT 828 showed similar susceptibility patterns against the same analogs, although the MICs of these compounds were between 25 and 50 µg/mL against this strain. In contrast, *B. cereus* UJA 27q was the most resistant strain, being susceptible to just three of the analogs tested.

### 2.3. Antibiofilm Activity

#### 2.3.1. Inhibition of Biofilm Formation

Based on the previous results of antibacterial activity, strains *S. aureus* CECT 976, *S. aureus* CECT 828 and *B. cereus* UJA 27q along with the nine compounds that showed the best antibacterial activity were selected for analyzing their ability to inhibit the biofilm formation by these target strains (Table 3).

The best results (with more than 90% of biofilm inhibition by target bacteria) were found for compound **10** (97.9% of inhibition against *S. aureus* CECT 976, at a concentration of 0.1 µg/mL), compound **3** (97.2% of inhibition at 0.01 µg/mL against *B. cereus* UJA 27q), compound **1** (96.8% of inhibition at 0.1 µg/mL against *S. aureus* CECT 976), compounds **8** (95.7% at 50 µg/mL) and **10** (94.2% at 25 µg/mL) against *S. aureus* CECT 828 and finally, compound **11** (93.9% of inhibition at 0.01 µg/mL) against *B. cereus* UJA 27q.

Taking into account their chemical structure, three of these especially active compounds (**1**, **3** and **8**) have a coumarin moiety. All of these compounds also have OH groups in their structure, which seems to be of great value to favor the ability to inhibit the biofilm formation by target bacteria. However, the efficacy of the compounds also depends on the target strain, so compounds **1** and **10** are the most active against *S. aureus* CECT 976, compounds **8** and **10** against *S. aureus* CECT 828 and compounds **3** and **11** against *B. cereus* UJA 27q, respectively (Table 3).

A second group of compounds, which showed biofilm inhibition of 65 to 90% on the selected target strains, includes compounds **9** at 10 µg/mL, compound **3** at 10 µg/mL and compound **6** at 25 µg/mL against *S. aureus* CECT 976 (with percentages of biofilm inhibition of 88.9%, 85.7% and 83.9%, respectively); compound **11** at 0.01 µg/mL against *S. aureus* CECT 828 (81.2% inhibition) and against *S. aureus* CECT 976 (77.6% of inhibition); compound **9** at 0.01 µg/mL and compound **7** at 1 µg/mL against *S. aureus* CECT 828 (71,25% and 69.9% of inhibition, respectively); compounds **8** at 25 µg/mL and **12** at 10 µg/mL against *S. aureus* CECT 976 (69.6% and 69.5% of inhibition) and at 0.01 µg/mL against *S. aureus* CECT 828 (68.46% of inhibition) and compound **10** at 0.01 µg/mL against *B. cereus* UJA 27q (68.2% oh inhibition). Similar results were previously found for analogs with a NO_2_ group at the A-ring against *B. cereus* UJA 27q [14].

Of the eight compounds that form this group, five of them, including the three with highest activity, have the coumarin moiety. Moreover, all of them excluding compound **6** have OH groups in their chemical structure, which increase their antibacterial activity as previously shown. In contrast, compound **6** has a Cl group, which favors the ability to inhibit biofilm formation by target cells.

The most susceptible strain in these assays was *S. aureus* CECT 976, being inhibited in the biofilm formation by six of the assayed compounds (**9**, **3**, **6**, **11**, **8** and **12**). Four compounds (**11**, **9**, **7**, **12**) were able to inhibit biofilm formation by *S. aureus* CECT 828 and just one compound (**10**) had the same effect on *B. cereus* UJA 27q, along with the previously studied analogs with a NO_2_ group at the A-ring [14].

Finally, less active compounds, with 50 to 65% of inhibition in biofilm formation by the target bacteria, were compound **6** at 10 µg/mL against *S. aureus* CECT 828 (51.9% of inhibition), compound **7** at 10 µg/mL against *S. aureus* CECT 976 (50.9% of inhibition) and compound **3** at 1 µg/mL against *S. aureus* CECT 828 (49.22% of inhibition). All these three compounds have the common coumarin moiety in their chemical structure.

In contrast, some compounds, such as **16** and **17**, increase the biofilm formation by *S. aureus* CECT 976, *S. aureus* CECT 828 and *B. cereus* UJA 27q.

**Figure 4:**
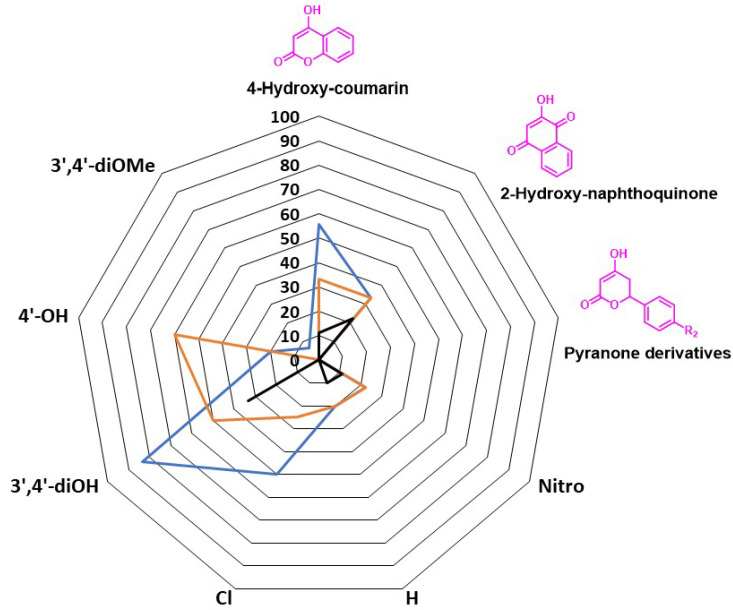
Structure–activity of analogs to A-type procyanidins (**1**–**24**) against the inhibition of biofilm of selected foodborne pathogens (*S. aureus* CECT 976, *S. aureus* CECT 828 and *B. cereus* UJA 27q). **Blue line**: number of compounds (expressed in percentage) with more than 65% of biofilm inhibition against *S. aureus* CECT 976. **Orange line**: number of compounds (expressed in percentage) with more than 65% of biofilm inhibition against *S. aureus* CECT 828. **Black line**: number of compounds (expressed in percentage) with more than 65% of biofilm inhibition against *B. cereus* UJA 27q.

Regarding the structure–activity relationships, compounds with a coumarin moiety show higher activity against *S. aureus* strains and those with a naphthoquinone moiety seem to be more active against *B. cereus* (Figure 4).

#### 2.3.2. Disruption of Preformed Biofilms

The ability of the analogs in disrupting previously formed biofilms by food pathogens was also evaluated (Table 3). Strains *S. aureus* CECT 976, *S. aureus* CECT 828 and *B. cereus* UJA 27q and the nine analogs with best activity were selected again to analyze the disruption of preformed biofilms. All these compounds were able to disrupt more than 50% of preformed biofilms for at least one of the assayed bacteria. Moreover, concentrations of 0.01 µg/mL or 0.1 µg/mL were enough to disrupt the biofilms, regardless of the assayed strain.

The best activity was observed for compounds **10** and **11**, both with a naphthoquinone moiety and with two OH groups in the molecule.

Compound **8** at 0.01 µg/mL also stands out, being able to disrupt 86.2% of the preformed biofilm by *S. aureus* CECT 976, together with compound **11** at 0.01 µg/mL against *B. cereus* UJA 27q, with disruptions of more than 75% of the preformed biofilms.

## 3. Discussion

Compared to previous results obtained using analogs with floroglucinol or resorcinol as the D–ring [13,14], higher antibacterial activities of these new analogs were detected, although analog **IV** described in [13] showed similar antibacterial activity against *B. cereus* UJA 27q compared to analogs **2** and **11** reported here. These results point to the nucleophilic unit used in the synthesis of A–type PC analogs as one of the most important aspects to consider when designing new antibacterials derived from A–type proanthocyanidins. In that sense, coumarin and/or naphthoquinone instead of fluoroglucinol, resorcinol and/or pyranone moieties significantly increase the antibacterial activity of the prepared compounds against these foodborne bacteria.

Our synthetic compounds showed higher antibacterial activities compared to natural products, as previously described for chlorinated thymol and carvracol derivatives on *S. aureus* and *P. aeruginosa* [18]. Similar results have also been previously described by us with phenolic compounds against these target strains. We tested six analogs of A–type proanthocyanidins that were able to inhibit the growth of twenty-one foodborne bacteria, as well as inhibit the biofilm formation and disrupt preformed biofilms by these target bacteria [13].

The hydroxylation at positions 5 and 7 on the A–ring has been previously described to play an important role in the antibacterial activity of flavonols [19]. Moreover, hydroxylations on the C–ring increased the activity. Therefore, the number of monomeric subunits and the location of B–ring hydroxyl groups of the flavan-3-ol monomer are important factors that define the chemistry and bioactivities of condensed tannins (CTs) [20]. Our results also corroborate these previously established structure–activity relationships, so the presence of OHs at the B–ring and halogenated atoms like Cl at the A–ring provide higher antibacterial activities [14,15].

Regarding antibiofilm activity of our compounds, various biofilm inhibitory mechanisms have been reported for CT, such as bacterial growth reduction properties, bacterial membrane impairment, and inhibition of the production of an extracellular matrix against *P. aeruginosa* [21]. Cranberry proanthocyanidins have also showed antibiofilm properties against *P. aeruginosa* by down-regulating the expression of the citric acid cycle and ATP synthesis proteins in bacterial metabolism [22], and CT from astringent persimmons showed anti-biofilm activity against intraoral bacteria by reducing the hydrophobicity of bacteria [23]. The proanthocyanidins from highbush blueberry are also able to inhibit biofilm formation by altering the cell membrane integrity [24]. In contrast, the increase in biofilm formation by *S. aureus* CECT 976, *S. aureus* CECT 828 and *B. cereus* UJA 27q induced by compounds **16** and **17** is probably due to the capacity of some phenolic compounds to induce partial bacterial lysis and subsequent aggregation and membrane fusion, which may favor biofilm formation [25]. Similar results have been previously detected and a paradoxical effect has been described in phenolic compounds against *E. coli* with cinnamtannin B–1 and against *Candida albicans* with proanthocyanidins [26] due to the association of some phenolic compounds to each other when the concentration increases [27] and because of the formation of aggregates with proteins and peptides [28], which reduces the effective concentration of these phenolic compounds.

Changes in exopolysaccharide (EPS) production or motility in both Gram–positive and Gram–negative bacteria, as well as changes in hydrophobicity, may also account for the antibiofilm activities we have found in our analogs, as previously described for some natural and derived compounds [29]. In general, tannin compounds act against bacteria, causing disintegration of bacterial colonies, by interfering with the bacterial cell wall and inhibiting fatty acid biosynthesis pathways [20]. They also act through iron chelation, damage to the cell membrane, inhibition of enzyme activities or interaction with proteins [30]. Interaction of tannins with cell wall synthesis also makes bacteria more susceptible to osmotic lysis and the alteration of the structure of the bacterial membranes may also increase fluidity, enhancing the effect of antibiotics [31]. Proanthocyanidins can also bind to the lipopolysaccharides of Gram–negative bacteria, leading to destabilization of the integrity of the outer membrane. They may also inhibit numerous bacterial enzymes, such as protease, phospholipase, urease, neuraminidase, and collagenase [32]. The expression and activity of the urease gene in *Proteus mirabilis* has also been described to be inhibited by tannic acid, with subsequent reduction in biofilm formation. Recent research efforts are addressing this gap, suggesting that PCs exert antibiofilm activities either by modulating quorum-sensing (QS) systems or by affecting elements such as the composition of the EPS matrix and bacterial motility [9], although further studies are necessary to corroborate this hypothesis.

In general, our compounds reported here present high antibacterial and antibiofilm activities specifically against three Gram–positive bacteria of great relevance for the food sector, including two strains of *S. aureus*, which are widely studied as a target of phenolic compounds, mainly because of the high virulence in methicillin–resistant *S. aureus* (MRSA) and their capacity to cause recurrent and durable infections in humans [33]. However, wider studies including a panel of reference strains of bacteria should be conducted in order to contribute to the active development of new food packaging preventing contamination by foodborne pathogens, lengthening the food shelf life and increasing the options of additives that can be used industrially.

## 4. Materials and Methods

### 4.1. General Experimental Methods

The solvents and reagents used, reactions performed and instrumentation were reported by us in a previous work [16]. In brief, all reactions were performed under inert atmospheric conditions at either room temperature or 50 °C. Reaction progress was monitored using analytical thin-layer chromatography (TLC) on silica gel 60 F254 precoated aluminum sheets (0.25 mm, Merck Chemicals, Darmstadt, Germany), with visualization achieved under ultraviolet light at 254 nm. Purification of the synthesized compounds was carried out via column chromatography (CC) using silica gel 60 (particle size 0.040–0.063 mm, Merck Chemicals, Darmstadt, Germany).

Nuclear magnetic resonance (NMR) spectra, including ^1^ H and ^13^ C, were recorded on a Bruker Avance 400 spectrometer (Bruker Daltonik GmbH, Bremen, Germany) operating at 400 MHz for ^1^ H and 100 MHz for ^13^ C. Deuterated solvents such as methanol (CD_3_OD), chloroform (CDCl_3_), and dimethyl sulfoxide ((CD_3_)_2_SO) were used, with a drop of deuterated trifluoroacetic acid (TFA-*d*) added for flavylium salts to establish acidic conditions. High-resolution mass spectra (HRMS) were obtained using an Agilent 6520B Quadrupole Time-of-Flight (QTOF) mass spectrometer (Agilent Technologies, Santa Clara, CA, USA).

#### 4.1.1. General Procedure for the Synthesis of Flavylium Salts

In a round-bottom flask, a salicylaldehyde derivative (1 mmol), an acetophenone derivative (1 mmol), concentrated H_2_SO_4_ (0.3 mL, 5.4 mmol), and acetic acid (HOAc, 1.3 mL) were combined. The mixture was stirred at room temperature overnight, as previously described [16]. Subsequently, diethyl ether (Et_2_O, 20 mL) was gradually added, resulting in the precipitation of a reddish solid. The solid was collected by filtration, thoroughly washed with additional diethyl ether, and dried. The synthesized flavylium salts were consistent with our prior reports, yielding comparable efficiencies, and their structures were confirmed by comparison to previously reported spectral data [16].

#### 4.1.2. General Procedure for the Synthesis of 2,8-Dioxabicyclo [3.3.1] Nonanes (**1**–**24**)

In a round-bottom flask, a flavylium salt (0.5 mmol) was reacted with 4-hydroxycoumarin, 2-hydroxy-1,4-naphthoquinone, 4-hydroxy-6-phenyl-5,6-dihydro-2H-pyran-2-one, 6-(4-chlorophenyl)-4-hydroxy-5,6-dihydro-2H-pyran-2-one, or 4-hydroxy-6-(4-methoxyphenyl)-5,6-dihydro-2H-pyran-2-one (0.5 mmol) in absolute methanol (MeOH, 8 mL). The reaction mixture was stirred at 50 °C overnight in an oil bath, as previously detailed [16]. Following the reaction, the solvent was evaporated, and the resulting crude product was purified using column chromatography with silica gel 60 as the stationary phase. The synthesized dioxabicyclic derivatives (**1**–**24**) were consistent with our previous reports [16], and their structures were confirmed by comparison with previously reported spectral data [16].

### 4.2. Antibacterial Activity

With the aim to estimate the efficacy of the compounds against different foodborne bacteria, their antibacterial and antibiofilm activities were evaluated. The compounds were dissolved in dimethyl sulfoxide (DMSO) (Sigma-Aldrich, Madrid, Spain) and serially diluted for antimicrobial and antibiofilm assays. All experiments were carried out in triplicate.

Preliminary studies on the antibacterial activity of the compounds were performed by the standard agar diffusion method as previously described [15]. Next, minimal inhibitory concentration (MIC) values for each compound were obtained by the broth microdilution method, according to the recommendations of the CLSI (2015) [34]. Briefly, serial dilutions of the compounds in tryptic soya broth (TSB) (Scharlab, Barcelona, Spain) were incubated with bacterial suspensions of the target strains (10^5^ CFU in TSB) during 24 h, at 37 °C. Then, plates were read on an iMarkMicroplate Reader (Bio-Rad, Madrid, Spain) at OD595 to determine the minimal concentration of each of the compounds tested being able to inhibit bacterial growth. As targets for these assays, both strains from Type-Culture Collections (Spanish; CECT and from the University of Goteborg; CCUG) as well as strains of our own collection from organic foods [35] were used (Table 4).

### 4.3. Biofilm Formation Inhibition Assay and Disruption of Preformed Biofilm

In order to detect antibiofilm activities of the compounds against the target strains, bacteria were incubated with 10-fold serially diluted purified compounds according to Ulrey et al. [22]. Bacterial suspensions (10^5^ CFU in TSB) were incubated with increasing concentrations of each compound, (24 h, 30 °C). Wells with bacterial suspensions and TSB medium were run in parallel as positive controls for biofilm formation. All wells were washed with tap water, and the biofilms fixed with methanol. The plate was stained with 0.3% crystal violet and read on an iMarkMicroplate Reader (Bio-Rad, Madrid, Spain) OD595.

The ability of the analogs in disrupting previously formed biofilms by food pathogens may also be of great interest for food industries, so cells were then allowed to form biofilms during 24 h in a subsequent assay, and once the bacteria had formed these structures, diluted compounds were added to the plates in order to detect the remaining biofilm after a second incubation (24 h, 30 °C) by the crystal violet stain method.

### 4.4. Statistical Analysis

The average data and standard deviations from absorbances of antibiofilm assays were determined with the Excel program version 18.0 (Microsoft Corp., Redmond, WA, USA). The statistical significance of the data was evaluated by a *t*-test that was performed at the 95% confidence level with Statgraphics Plus version 5.1 (Statistical Graphics Corp., Rockville, MD, USA).

## 5. Conclusions

*S. aureus* CECT 976, *S. aureus* CECT 828 and *B. cereus* UJA27q were the most susceptible strains with regard to both antibacterial and antibiofilm activities when faced with most of the analyzed compounds. Regarding the structure–activity relationships observed, the coumarin nucleophilic unit seems to favor the antibacterial activity against both *S. aureus* strains, while a naphthoquinone moiety enhances antibacterial effects against *B. cereus* UJA27q. Moreover, the replacement of OH groups in the B-ring by methoxy groups (compounds **4**, **5**, **6**, **13**, **14**, **15** and **19a** to **24b**) impairs the antibacterial activity of the compounds against target bacteria, while the presence of Cl or OH groups in the molecules seems to enhance the inhibition of biofilm formation as well as the disruption of preformed biofilms.

## Figures and Tables

**Table 1:** Growth inhibition concentration (µg/mL) of analogs against target strains.

Compound	*S. aureus* CECT 976	*S. aureus* CECT 828	*L. innocua* CECT 910	UJA27q	UJA27t	UJA11c	UJA40k	UJA34f
**1**	100	1000		1000				
**3**	100	100		100				
**6**	100	1000						1000
**7**	100	100						1000
**8**	100	100						100
**9**	10	100	100					100
**10**	100	100		100				1000
**11**	100	100		100				1000
**12**	100	100	100					1000
**16**	10	100	100	100	100			1000
**17**	10	100	100	100		1000	100	1000

**Table 2:** MICs of analogs against target strains (µg/mL).

Compound	*S. aureus* CECT 976	*S. aureus* CECT 828	UJA27q
**1**	25		
**3**	25	25	25
**6**	25	50	
**7**	25	25	
**8**	25	50	
**9**	25	25	
**10**	25	50	25
**11**	50	25	25
**12**	50	50	

**Table 3:** Inhibition of biofilm and disruption of preformed biofilm by analogs against target strains.

Compound	Concentration (µg/mL)	Inhibition of Biofilm (%)			Disruption of Preformed Biofilm (%)		
		*S. aureus* CECT 976	*S. aureus* CECT 828	UJA27q	*S. aureus* CECT 976	*S. aureus* CECT 828	UJA27q
**1**	0.1	96.85 ± 8.38 **					
	0.01				61.22 ± 4.66 *		
**3**	10	85.71± 7.12 *					
	1		49.22 ± 3.36				
	0.1					71.29 ± 7.44 *	
	0.01			97.22 ± 10.21 **			54.08 ± 6.58
**6**	25	83.93 ± 8.87 *					
	10		51.87 ± 3.12				
	0.01				76.44 ± 6.98 *	59.3 ± 6.45	
**7**	10	50.87 ± 4.79			77.1 ± 8.12 *		
	1		69.89 ± 5.79 *				
	0.1					72.6 ± 6.95 *	
**8**	50		95.69 ± 9.45 **				
	25	69.58 ± 7.01 *					
	0.01				86.2 ± 7.69 *	77.44 ± 6.13 *	
**9**	10	88.9 ± 7.58 *			66.23 ± 5.89 *		
	0.01		71.25 ± 6.36 *				
**10**	25		94.23 ± 10.12 **				
	0.1	97.99 ± 9.87 **					
	0.01			68.19 ± 6.11 *	76.5 ± 6.58 *	77.55 ± 4.37 *	72.96 ± 5.17 *
**11**	25	77.57 ± 6.96 *					
	0.1				66.4 ± 7.85		
	0.01		81.17 ± 7.18 *	93.94 ± 8.75 **		74.62 ± 6.58 *	83.5 ± 6.12 *
**12**	10	69.50 ± 5.22 *					
	0.01		68.46 ± 3.41 *		65.39 ± 4.55 *	75.84 ± 6.16 *	

Biofilm persistence in the absence of compounds was considered as 100%; * *p* < 0.05; ** *p* < 0.01.

**Table 4:** Bacterial strains tested in antibacterial assays.

Strains from Type-Culture Collections	Resistant Strains from Organic Foods
*Salmonella enterica* CECT 915	*Bacillus cereus* UJA27q
*Salmonella enterica* CECT 4300	*Enterococcus casseliflavus* UJA11e
*Escherichia coli* CCUG 47553	*Enterococcus faecium* UJA11c
*Escherichia coli* CCUG 47557	*Staphylococcus aureus* UJA34f
*Staphylococcus aureus* CECT 828	*Staphylococcus saprophyticus* UJA27g
*Staphylococcus aureus* CECT 976	*Lactobacillus casei* UJA35h
*Staphylococcus aureus* CECT 4465	*Enterobacter* sp. UJA37p
*Listeria monocytogenes* CECT 4032	*Pantoea agglomerans* UJA7m
	*Pantoea agglomerans* UJA29o
	*Klebsiella terrigena* UJA32j
	*Salmonella* sp. UJA40k
	*Salmonella* sp. UJA40l

## Data Availability

Data are contained within the article and Supplementary Materials.

## Supplementary Materials

The following supporting information can be downloaded at https://www.mdpi.com/article/10.3390/molecules30040944/s1: Table S1: Systematic names of synthesized compounds (**1**–**24**).
