# Supplementary material for: Evaluation of Antibacterial and Antibiofilm Properties of Phenolics with Coumarin, Naphthoquinone and Pyranone Moieties Against Foodborne Microorganisms
Source: Molecules. 2025 Feb 18;30(4):944. doi: 10.3390/molecules30040944 (PMC11857956; doi:10.3390/molecules30040944)
Supplement: Supplementary file 1 [file molecules-30-00944-s001.zip › molecules-3423452-supplementary.pdf]

SUPPLEMENTARY MATERIAL

Evaluation of Antibacterial and Antibiofilm Properties of Phenolics with Coumarin, Naphthoquinone and Pyranone Moieties Against Foodborne Microorganisms

Alejandra Alejo-Armijo [1], Antonio Cobo [1,†], Alfonso Alejo-Armijo [2,*,‡], Joaquín Altarejos [2], Sofía Salido [2] and Elena Ortega-Morente [1,*]

1 Department of Health Sciences, Faculty of Experimental Sciences, University of Jaén, Campus of International Excellence in Agri-Food (ceiA3), 23071 Jaén, Spain; ajanja00@gmail.com (A.A.-A.); acmolinos@ugr.es (A.C.)
2 Department of Inorganic and Organic Chemistry, Faculty of Experimental Sciences, University of Jaén, Campus of International Excellence in Agri-Food (ceiA3), 23071 Jaén, Spain; jaltare@ujaen.es (J.A.); ssalido@ujaen.es (S.S.)
* Correspondence: aalejo@ujaen.es (A.A.-A.); eortega@ujaen.es (E.O.-M.)
† Current address: Departamento de Microbiología, Universidad de Granada, 18071 Granada, Spain
‡ Current address: Departamento de Ciencias Farmacéuticas, Facultad de Farmacia, Universidad de Salamanca, Campus Miguel de Unamuno, 37007 Salamanca, Spain.

Table S1. Systematic names of synthesized compounds (**1**–**24**)

Compound	Name
**1**	8-(3,4-dihydroxyphenyl)-1*H*,14*H*-8,14-methanobenzo[7,8][1,3]dioxocino[5,4-*c*]chromen-1-one
**2**	8-(3,4-dihydroxyphenyl)-12-nitro-1*H*,14*H*-8,14-methanobenzo[7,8][1,3]dioxocino[5,4-*c*]chromen-1-one
**3**	12-chloro-8-(3,4-dihydroxyphenyl)-1*H*,14*H*-8,14-methanobenzo[7,8][1,3]dioxocino[5,4-*c*]chromen-1-one
**4**	8-(3,4-dimethoxyphenyl)-1*H*,14*H*-8,14-methanobenzo[7,8][1,3]dioxocino[5,4-*c*]chromen-1-one
**5**	8-(3,4-dimethoxyphenyl)-12-nitro-1*H*,14*H*-8,14-methanobenzo[7,8][1,3]dioxocino[5,4-*c*]chromen-1-one
**6**	12-chloro-8-(3,4-dimethoxyphenyl)-1*H*,14*H*-8,14-methanobenzo[7,8][1,3]dioxocino[5,4-*c*]chromen-1-one
**7**	8-(4-hydroxyphenyl)-1*H*,14*H*-8,14-methanobenzo[7,8][1,3]dioxocino[5,4-*c*]chromen-1-one
**8**	8-(4-hydroxyphenyl)-12-nitro-1*H*,14*H*-8,14-methanobenzo[7,8][1,3]dioxocino[5,4-*c*]chromen-1-one
**9**	12-chloro-8-(4-hydroxyphenyl)-1*H*,14*H*-8,14-methanobenzo[7,8][1,3]dioxocino[5,4-*c*]chromen-1-one
**10**	6-(3,4-dihydroxyphenyl)-13*H*-6,14-methanobenzo[*d*]naphtho[2,3-*g*][1,3]dioxocine-8,13(14*H*)-dione
**11**	6-(3,4-dihydroxyphenyl)-2-nitro-13*H*-6,14-methanobenzo[*d*]naphtho[2,3-*g*][1,3]dioxocine-8,13(14*H*)-dione
**12**	2-chloro-6-(3,4-dihydroxyphenyl)-13*H*-6,14-methanobenzo[*d*]naphtho[2,3-*g*][1,3]dioxocine-8,13(14*H*)-dione
**13**	6-(3,4-dimethoxyphenyl)-13*H*-6,14-methanobenzo[*d*]naphtho[2,3-*g*][1,3]dioxocine-8,13(14*H*)-dione
**14**	6-(3,4-dimethoxyphenyl)-2-nitro-13*H*-6,14-methanobenzo[*d*]naphtho[2,3-*g*][1,3]dioxocine-8,13(14*H*)-dione
**15**	2-chloro-6-(3,4-dimethoxyphenyl)-13*H*-6,14-methanobenzo[*d*]naphtho[2,3-*g*][1,3]dioxocine-8,13(14*H*)-dione
**16**	6-(4-hydroxyphenyl)-2-nitro-13*H*-6,14-methanobenzo[*d*]naphtho[2,3-*g*][1,3]dioxocine-8,13(14*H*)-dione
**17**	2-chloro-6-(4-hydroxyphenyl)-13*H*-6,14-methanobenzo[*d*]naphtho[2,3-*g*][1,3]dioxocine-8,13(14*H*)-dione
**18**	(3R,6R,12*S*)-6-(3,4-dimethoxyphenyl)-3-phenyl-4,12-dihydro-1*H*,3*H*-6,12-methanobenzo[*d*]pyrano[3,4-*g*][1,3]dioxocin-1-one + enantiomer
**19a**	(3*S*,6*R*,12*S*)-3-(4-chlorophenyl)-6-(3,4-dimethoxyphenyl)-4,12-dihydro-1*H*,3*H*-6,12-methanobenzo[*d*]pyrano[3,4-*g*][1,3]dioxocin-1-one + enantiomer
**19b**	(3*R*,6*R*,12*S*)-3-(4-chlorophenyl)-6-(3,4-dimethoxyphenyl)-4,12-dihydro-1*H*,3*H*-6,12-methanobenzo[*d*]pyrano[3,4-*g*][1,3]dioxocin-1-one + enantiomer
**20a**	(3*S*,6*R*,12*S*)-6-(3,4-dimethoxyphenyl)-3-(4-methoxyphenyl)-4,12-dihydro-1*H*,3*H*-6,12-methanobenzo[*d*]pyrano[3,4-*g*][1,3]dioxocin-1-one + enantiomer
**20b**	(3*R*,6*R*,12*S*)-6-(3,4-dimethoxyphenyl)-3-(4-methoxyphenyl)-4,12-dihydro-1*H*,3*H*-6,12-methanobenzo[*d*]pyrano[3,4-*g*][1,3]dioxocin-1-one + enantiomer
**21a**	(3*S*,6*R*,12*S*)-6-(3,4-dimethoxyphenyl)-10-nitro-3-phenyl-4,12-dihydro-1*H*,3*H*-6,12-methanobenzo[*d*]pyrano[3,4-*g*][1,3]dioxocin-1-one + enantiomer
**21b**	(3*R*,6*R*,12*S*)-6-(3,4-dimethoxyphenyl)-10-nitro-3-phenyl-4,12-dihydro-1*H*,3*H*-6,12-methanobenzo[*d*]pyrano[3,4-*g*][1,3]dioxocin-1-one + enantiomer
**22**	(3*R*,6*R*,12*S*)-3-(4-chlorophenyl)-6-(3,4-dimethoxyphenyl)-10-nitro-4,12-dihydro-1*H*,3*H*-6,12-methanobenzo[*d*]pyrano[3,4-*g*][1,3]dioxocin-1-one + enantiomer
**23**	(3*R*,6*R*,12*S*)-10-chloro-3-(4-chlorophenyl)-6-(3,4-dimethoxyphenyl)-4,12-dihydro-1*H*,3*H*-6,12-methanobenzo[*d*]pyrano[3,4-*g*][1,3]dioxocin-1-one
**24**	(3*R*,6*R*,12*S*)-10-chloro-6-(3,4-dimethoxyphenyl)-3-(4-methoxyphenyl)-4,12-dihydro-1*H*,3*H*-6,12-methanobenzo[*d*]pyrano[3,4-*g*][1,3]dioxocin-1-one
